# Age Differences in the Affective Experience of State Curiosity

**DOI:** 10.1093/geroni/igaa057.1472

**Published:** 2020-12-16

**Authors:** Li Chu, Helene Fung

**Affiliations:** 1 The Chinese University of Hong Kong, Shatin, New Territories, China; 2 The Chinese University of Hong Kong, Sha Tin, China

## Abstract

Is feeling curious a pleasant, anxious or mixed feeling experience? Dual process theory posits that curiosity results from an optimal level of knowledge gap anxiety. Yet, personal growth facilitation model suggests that people are intrinsically curious, which is associated with positive affects. While curiosity may be pleasant or anxious, it may also be both. In fact, compared with younger adults, older adults were more likely to experience mixed emotions. However, very few studies investigated age differences in affective experience of curiosity, so the present study utilized a time-sampling dataset to address this question. This 14-day time-sampling study included 85 younger (43 females, age 18-30) and 83 older adults (40 females, age 60-85) who recorded momentary curiosity and affective experiences three times per day. Linear mixed-effects analysis revealed a significant 3-way interaction between age group, happiness and anxiousness on state curiosity (□=.20, SE=.05, p<.001). For younger adults, results suggested that curiosity was higher when they felt either happy or anxious but not when feeling both. Conversely, for older adults, curiosity was higher when they felt both happy and anxious concurrently. In other words, older adults were more likely to experience curiosity as a mixed emotional state, whereas younger adults were more likely to experience curiosity as a pure emotional state. This finding adds to the current mixed emotion and aging literature and has important implications for future interventions to enhance curiosity towards novelties for people from different age groups.

